# Impact of re-operation on progression-free survival in patients with recurrent GBM: Experience in a tertiary referral center

**DOI:** 10.1371/journal.pone.0317937

**Published:** 2025-01-31

**Authors:** Houssein Darwish, Tasnim Diab, Sarah Kawtharani, Mounir Barake, Bader Ali, Nagham Ramadan, Hiba Fadlallah, Jeannot Kekedjian, Marwan Najjar, Hazem I. Assi

**Affiliations:** 1 Department of Surgery, Division of Neurosurgery, American University of Beirut Medical Center, Beirut, Lebanon; 2 Department of Internal Medicine, Division of Hematology and Oncology, Naef K Bassile Cancer Institute, American University of Beirut Medical Center, Beirut, Lebanon; 3 Faculty of Medicine, American University of Beirut, Beirut, Lebanon; Goethe University Hospital Frankfurt, GERMANY

## Abstract

**Background:**

Reoperation for patients with recurrent glioblastoma multiforme (GBM) is a highly debated topic within the medical community. GBM is known for its aggressive nature and poor prognosis, with most patients experiencing tumor recurrence despite initial treatments. Some studies suggest a survival benefit from a second surgery, while others do not. The aim of this study is to assess whether reoperation for recurrent GBM offers a survival benefit compared to patients who do not undergo re-resection and to identify the prognostic factors influencing patient selection for reoperation.

**Methods:**

This study retrospectively reviewed medical records from the American University of Beirut Medical Center over a ten-year period, from 01/01/2012 to 01/01/2023. It included patients with recurrent GBM after initial surgical resection. Patients were categorized into two groups: those who underwent reoperation and those who received only medical management upon recurrence. Inclusion criteria included histologically confirmed GBM with previous tumor resection; patients who only had a biopsy were excluded. Time to progression and time to death were analyzed using the Kaplan-Meier curve, with differences between groups assessed by the log-rank test.

**Results:**

Age categorization (≤50 vs. >50 years) and gender distribution did not significantly impact reoperation likelihood (p = 0.306 and p = 0.616, respectively). However, a notable association was observed with Charlson comorbidity index (CCI) ≤3, indicating higher reoperation rates (p = 0.022). Tumor size grouping (≤5 vs. >5 cm) showed no significant association with reoperation status (p = 0.175). Similarly, whether the tumor was unifocal or multifocal and the extent of initial tumor resection (GTR vs. subtotal) did not demonstrate significant associations with reoperation (p = 0.086 and p = 0.351, respectively). Remarkably, complications following the initial surgery emerged as a significant factor associated with the decision not to undergo reoperation (p = 0.018). The most common complications following both initial and subsequent surgeries included DVT, weakness, seizures, and wound leakage and infection. The progression-free survival for patients who underwent reoperation was 15.9 months, whereas for those who did not undergo reoperation, it was 6.7 months (log-rank p < 0.001) The median post progression survival for patients who underwent reoperation upon recurrence was 5.9 months, compared to 5.1 months for those who did not undergo reoperation. (log-rank p = 0.065). The median overall survival for patients who did not undergo reoperation was 11 months, compared to 21 months for those who underwent reoperation (log-rank p < 0.001).

**Conclusion:**

In conclusion, reoperation for recurrent Glioblastoma Multiforme (GBM) appears to offer a survival benefit, as indicated by significantly longer disease-free intervals and higher progression-free and overall survival rates compared to patients who did not undergo reoperation.

## Introduction

Glioblastoma multiforme (GBM) is the deadliest and most prevalent form of primary brain tumors in adults. According to the 2021 WHO Classification of Tumors of the Central Nervous System, glioblastoma is classified as a WHO grade 4 glioma based on molecular and histopathological features. It represents approximately 47.1% of all malignant tumors of the central nervous system (CNS), making it the most common malignant brain tumor [1]. Its global incidence rate stands at approximately 3–4/100,000 person-years, although regional variability exists [2].

The cornerstone of initial therapy for glioblastoma multiforme (GBM) is surgical resection, followed by adjuvant radiotherapy and temozolomide therapy, with additional options including immunotherapy or anti-angiogenic therapy. However, despite the aggressiveness of these regimens, their limited efficacy underscores its grim prognosis [3]. GBM is characterized by its continuous progression, a hallmark that prompted the development of standardized criteria for its assessment [1, 4, 5]. The Macdonald Criteria [6], introduced to provide a uniform method of evaluation, defines progression as a 25% increase in the sum of new lesions, perpendicular diameters of enhancing lesions, and clinical deterioration. The Response Assessment in Neuro-Oncology (RANO) criteria [7] have since evolved to offer an updated framework, emphasizing significant increases in non-enhancing lesions, FLAIR, as well as recognizing the insufficiency of post-contrast enhancement alone due to the infiltrative nature of GBM.

Despite current treatments, the median progression-free survival (PFS) remains relatively short, ranging from 4.4 to 8.4 months in newly diagnosed GBM cases [1, 4, 5]. To improve outcomes, novel therapeutic strategies have been developed for recurrent cases, including new targeted therapies, angiogenesis inhibitors, and gamma knife surgery [8, 9]. Recent studies have identified a range of clinical and molecular variables that significantly influence survival and recurrence in glioblastoma. Factors such as patient age, extent of surgical resection, molecular markers like MGMT methylation status, and the presence of genetic mutations have been shown to impact both progression-free survival and overall survival in glioblastoma patients [10].

Surgical approaches have also become a more frequently utilized option and are utilized in 10–30% of GBM cases [11]. Reoperation for recurrent glioblastoma was pioneered by Pool [12] in 1968. However, only a limited number of studies have examined its impact on glioblastoma recurrence. Nonetheless, reoperation has shown promise in improving PFS and overall survival in select patients. In addition, published evidence, particularly from Berger et al. [8] and other researchers, indicates similar outcomes [13, 14]. For patients undergoing repeat surgery, the NIH Recurrent Glioblastoma Scale offers a means to stratify outcomes. In the most favorable scenario, the median survival reached 9.2 months, contrasting sharply with the poorest-performing group, which experienced a median survival of only 1.9 months [15]. Overall, re-do craniotomy was shown to have enhanced survival outcomes. However, conducting randomized studies on reoperation is challenging due to the heterogenetic nature of recurrent GBM.

Furthermore, emerging research has highlighted the role of tumor stem cell dissemination in glioblastoma, particularly following ventricular opening during surgical resection. This process may contribute to the aggressive nature of glioblastoma recurrence, as the migration of tumor stem cells to distant sites within the brain increases the likelihood of tumor regrowth and progression after surgery. Such findings suggest that strategies targeting tumor stem cell spread could offer potential avenues for improving treatment efficacy in glioblastoma patients [16].

Additionally, the existing literature is predominantly retrospective, leading to potential confounding by selection bias. There is still some controversy on whether or not re-operation should be performed in patients with recurrent GBM, if this will offer any benefit regarding PFS, and, if any, what factors are correlated with significant benefit upon re-operation. Therefore, reoperation remains a topic of debate, particularly based on its applications and effectiveness [14]. Herein, we present a literature review on the matter while also presenting our data on patients with recurrent GBM in a tertiary referral center.

## Methods

This study retrospectively reviewed medical records from the American University of Beirut Medical Center (AUBMC) over a ten-year period, from 01/01/2012 to 01/01/2023. The data for this study were accessed from 04/04/2023 to 03/04/2024, inclusive. The study was conducted in accordance with the ethical principles outlined in the Declaration of Helsinki (2013) and its later amendments or equivalent ethical guidelines. Approval was granted by the Institutional Review Board (IRB) at AUBMC (study protocol ID: BIO-2023-0075) prior to data collection. All data were anonymized to ensure confidentiality, and the authors did not have access to any information that could identify individual participants during or after data collection. The information collected was fully anonymous, serially coded, and will remain confidential after the study concludes.

### Inclusion criteria

This study included patients diagnosed with recurrent glioblastoma multiforme (GBM) following initial surgical resection, with histologically confirmed GBM, classified according to the 2021 WHO Classification of Tumors of the Central Nervous System. While the 2021 classification was used for analysis, IDH-mutant GBM cases were not reclassified from the initial diagnoses based on the 2016 classification.

Only patients who underwent a single reoperation for recurrence were included; those with multiple resections were excluded. Tumors located in midline, thalamic, or infratentorial areas were excluded from the analysis. Patients who had undergone only biopsy were also excluded. Recurrence or progression was evaluated according to the Response Assessment in Neuro-Oncology (RANO) criteria, with pseudo-progression and necrosis excluded by MRI spectroscopy. Two groups were analyzed: those who underwent reoperation and those who received medical management upon recurrence.

### Statistical analysis

Continuous variables were described as means ± standard deviations or medians (interquartile range), and categorical variables were reported as frequencies and percentages. For inferential statistics, we evaluated significant associations with the decision to undergo reoperation using the Chi-square test for categorical variables and the t-test for continuous variables. All p-values were two-sided, and a significance level of p < 0.05 was applied to all analyses. The statistical analyses were performed using the SPSS version 29.0 statistical package.

### Survival analysis

Time to progression and overall survival were analyzed using the Kaplan-Meier method, with differences between groups assessed by the log-rank test. As a secondary objective, we performed a univariable Kaplan-Meier analysis to identify predictors of poor overall survival in patients with recurrent GBM. We also conducted a multivariable Cox regression analysis to assess independent predictors of poor overall survival in this cohort.

## Results

### Patient and tumor characteristics

A total of 243 patients were initially identified. However, 34 patients who had undergone only a biopsy without subsequent resection and 84 patients who had undergone surgical resection at our institution without follow-up on recurrence were excluded. Finally, data from 125 patients were included in our analysis. Of these, 84 patients had undergone a single resection, and 41 patients had undergone repeat resection **(**Table 1**)**.

**Table 1:** Patient and tumor characteristics.

	Single resection	Repeat resection	P-value
	N (%)	N (%)	
**Age**	53.11 ± 18.5	50.15 ± 13.4	0.363
**Gender**			
Male	61 (72.6)	28 (68.3)	0.616
Female	23 (27.4)	13 (31.7)	
**Charlson Comorbidity Index**			
≤ 3	37 (44)	27 (65.9)	**0.022**
> 3	47 (56)	14 (34.1)	
**ASA Classification**			
< 3	54 (71.1)	26 (70.3)	0.932
≥ 3	22 (28.9)	11 (29.7)	
**Tumor Location (Lobes)**			
Temporal	34 (40.5)	13 (31.7)	0.195
Parietal	18 (21.4)	15 (36.6)	
Frontal	23 (27.4)	12 (29.3)	
Occipital	3 (3.6)	1 (2.4)	
Other	6 (7.1)	0 (0)	
**Tumor Location (Side)**			
Right	37 (44)	18 (43.9)	1.000
Left	45 (53.6)	23 (56.1)	
Bilateral	2 (2.4)	0 (0)	
**Tumor Location**			
Eloquent	12 (14.3)	5 (12.2)	0.749
Non-Eloquent	72 (85.7)	36 (87.8)	
**Tumor Size**			
≤ 5 cm	47 (62.7)	15 (48.4)	0.175
< 5 cm	28 (37.3)	16 (51.6)	
**Tumor Focality**			
Unifocal	68 (81)	38 (92.7)	0.086
Multifocal	16 (19)	3 (7.3)	
**Extent of Tumor Resection**			
GTR	36 (46.2)	20 (55.6)	0.351
Subtotal	42 (53.8)	16 (44.4)	
**IDH Mutational Status**			
Wildtype	50 (87.7)	16 (72.7)	0.104
Mutant	7 (12.3)	6 (27.3)	
**Ki67 Index**			
High	77 (97.5)	28 (93.3)	0.303
Low	2 (2.5)	2 (6.7)	
**Oncotherapy after the 1^st^ Surgery**			
Resection only	5 (6)	1 (2.4)	0.681
RT alone	4 (4.8)	4 (9.8)	
Temodal or Avastin	1 (1.2)	0 (0)	
ChemoRT and Adj. CHT	73 (88)	36 (87.8)	
**Complications following 1^st^ Surgery**			
Yes	18 (21.4)	2 (4.9)	**0.018**
No	66 (78.6)	39 (95.1)	
**Location at Recurrence**			
Same	72 (93.5)	39 (97.5)	0.328
Different	5 (6.5)	1 (2.5)	

The mean age of patients who underwent single resection was 53.11 ± 18.5 years, compared to 50.15 ± 13.4 years for those who underwent repeat resection (p = 0.363). Gender distribution was similar across both groups, with males constituting 72.6% of the single resection cohort and 68.3% of the repeat resection cohort (p = 0.616).

### Influence of factors on the decision to undergo repeat resection

Analysis revealed that several factors significantly influenced the decision to pursue repeat resection. The Charlson Comorbidity Index showed a marked difference between the two groups. A higher proportion of patients undergoing repeat resection had a Charlson Comorbidity Index ≤ 3 (65.9%) compared to those undergoing single resection (44%) (p = 0.022). This suggests that patients with fewer comorbidities were more likely to undergo repeat resection.

Complications following the first surgery were notably less frequent in patients who chose to undergo repeat resection. Specifically, only 4.9% of patients who underwent repeat resection experienced complications following the initial surgery compared to 21.4% in the single resection group (p = 0.018). On multivariable analysis, complications following the first surgery emerged as the only significant factor associated with the decision not to undergo reoperation (p = 0.019).

There were no significant differences in tumor location distribution between both groups, and the temporal location was the most common in both groups (40.5% in single resection vs. 31.7% in repeat resection, p = 0.195). The tumor side was evenly distributed, with 44% of the single resection group and 43.9% of the repeat resection group having right-sided tumors (p = 1.000). Tumor size was also comparable between the groups, with 62.7% of the single resection group and 48.4% of the repeat resection group having tumors ≤ 5 cm (p = 0.175).

The extent of tumor resection revealed that 46.2% of the single resection group and 55.6% of the repeat resection group achieved gross total resection upon initial surgery (p = 0.351). The IDH mutational status was wildtype in 87.7% of the single resection group and 72.7% of the repeat resection group (p = 0.104). A high Ki67 index, typically considered above 20–30%, was found in 97.5% of the single resection group and 93.3% of the repeat resection group (p = 0.303).

Adjuvant treatment after the first surgery mainly consisted of chemoradiation and adjunctive chemotherapy (STUPP protocol), with 88% of patients in the single resection group and 87.8% in the repeat resection group (p = 0.681) undergoing oncotherapy after the first surgery. Upon recurrence, the tumor was found at the same site in 93.5% of patients in the single resection group and 97.5% of patients in the repeat resection group (p = 0.328).

### Complications following first surgery

The most common complications following the first surgery in the single resection group included deep vein thrombosis, hemiparesis/weakness, and seizures (Table 2). Other complications such as intracranial bleeding/stroke, wound infection, aphasia, facial palsy, and loss of vision were less frequent. In the repeat resection group, complications included deep vein thrombosis and seizures.

**Table 2:** Complications following first surgery: Single vs. repeat resection.

Complications following 1^st^ Surgery	Single resection	Repeat resection
	(Number of Patients)	(Number of Patients)
Deep vein thrombosis	6	1
Hemiparesis/ weakness	4	0
Seizures	2	1
Intracranial bleed/stroke	2	0
Wound infection	1	0
Aphasia	1	0
Facial palsy	1	0
Loss of vision	1	0

### Survival rates

Survival was analyzed across three key metrics: Overall Survival (OS), Post-Progression Survival (PPS), and Progression-Free Survival (PFS). OS was defined as the time from initial diagnosis to death or the end of follow-up, reflecting the length of time a patient lives after diagnosis. PPS was defined as the time from the first recurrence to death or the end of follow-up, indicating survival after the disease has recurred. PFS was defined as the time from initial diagnosis until disease progression or recurrence, capturing the duration of disease control before progression or recurrence.

**Fig 1:**
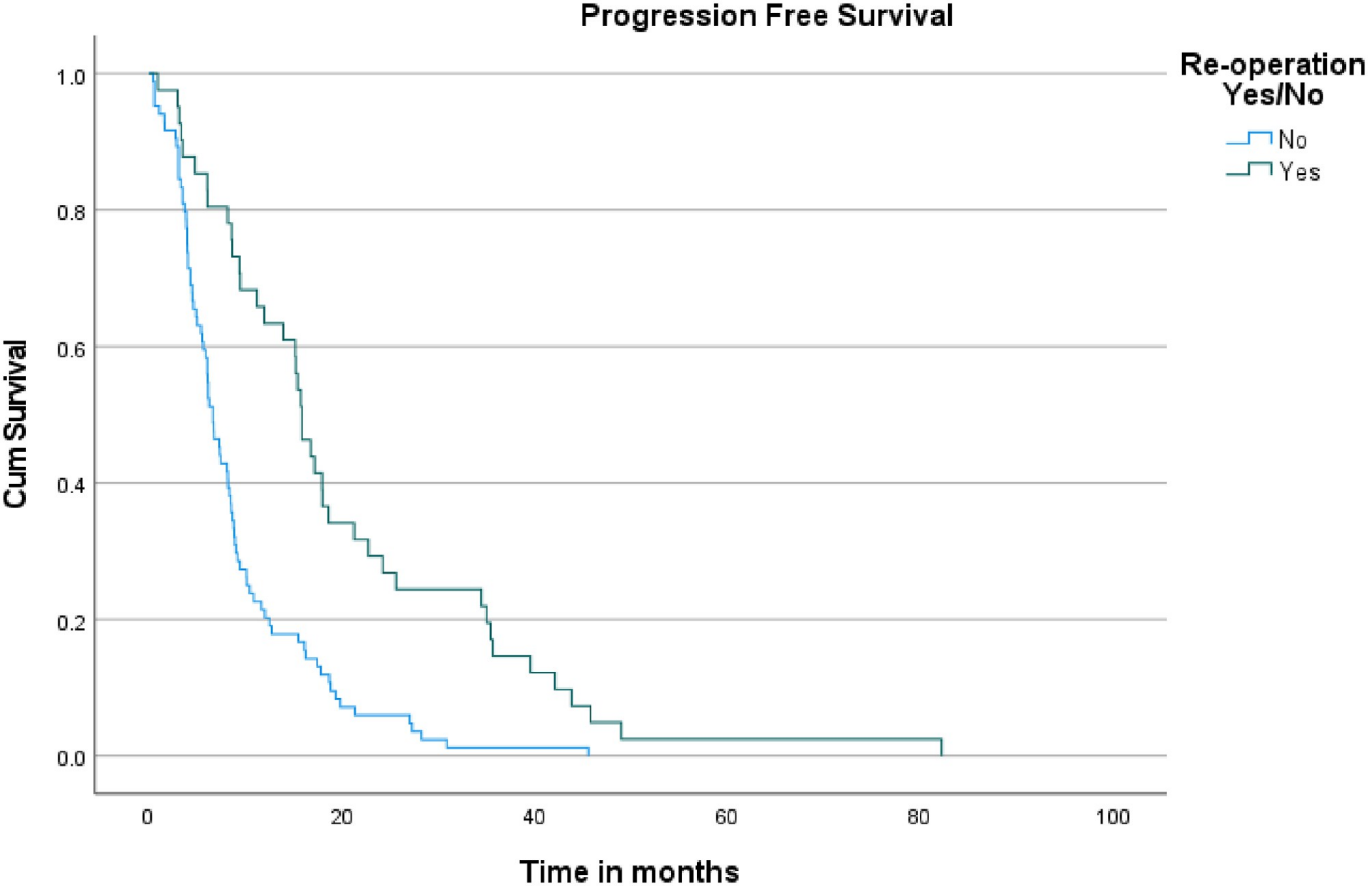
Kaplan-Meier curves for progression-free survival in patients undergoing initial resection vs. repeat resection.

Our analysis revealed significant findings related to reoperation. Patients who underwent reoperation had a median PFS of 15.9 months (95% CI: 13.906–17.828), compared to just 6.7 months (95% CI: 5.499–7.834) for those who did not undergo reoperation (log-rank p < 0.001) (Fig 1). This indicates that a longer PFS is associated with the decision to undergo repeat resection.

**Fig 2:**
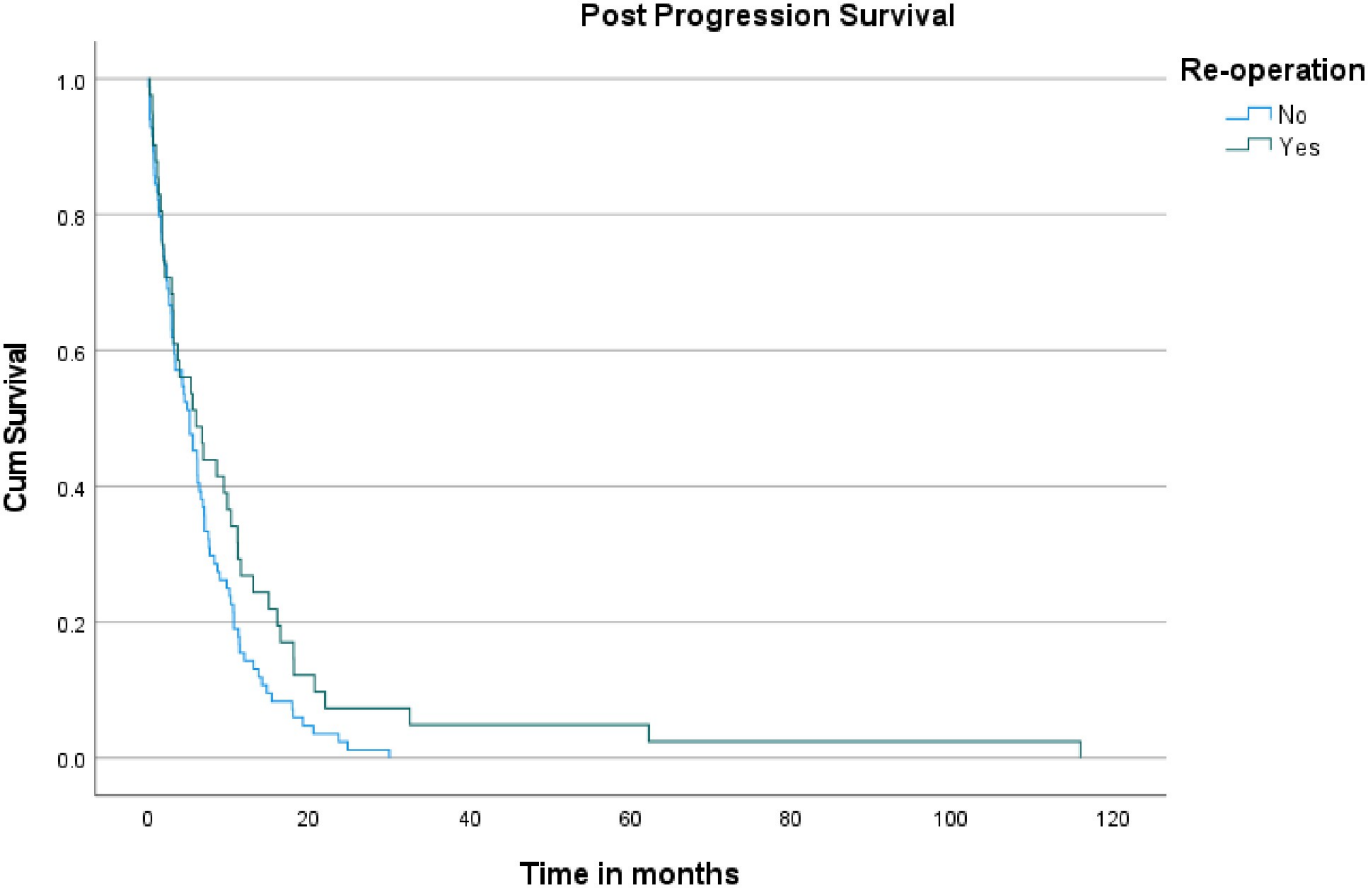
Kaplan-Meier curves for post-progression survival in patients undergoing initial resection vs. repeat resection.

**Fig 3:**
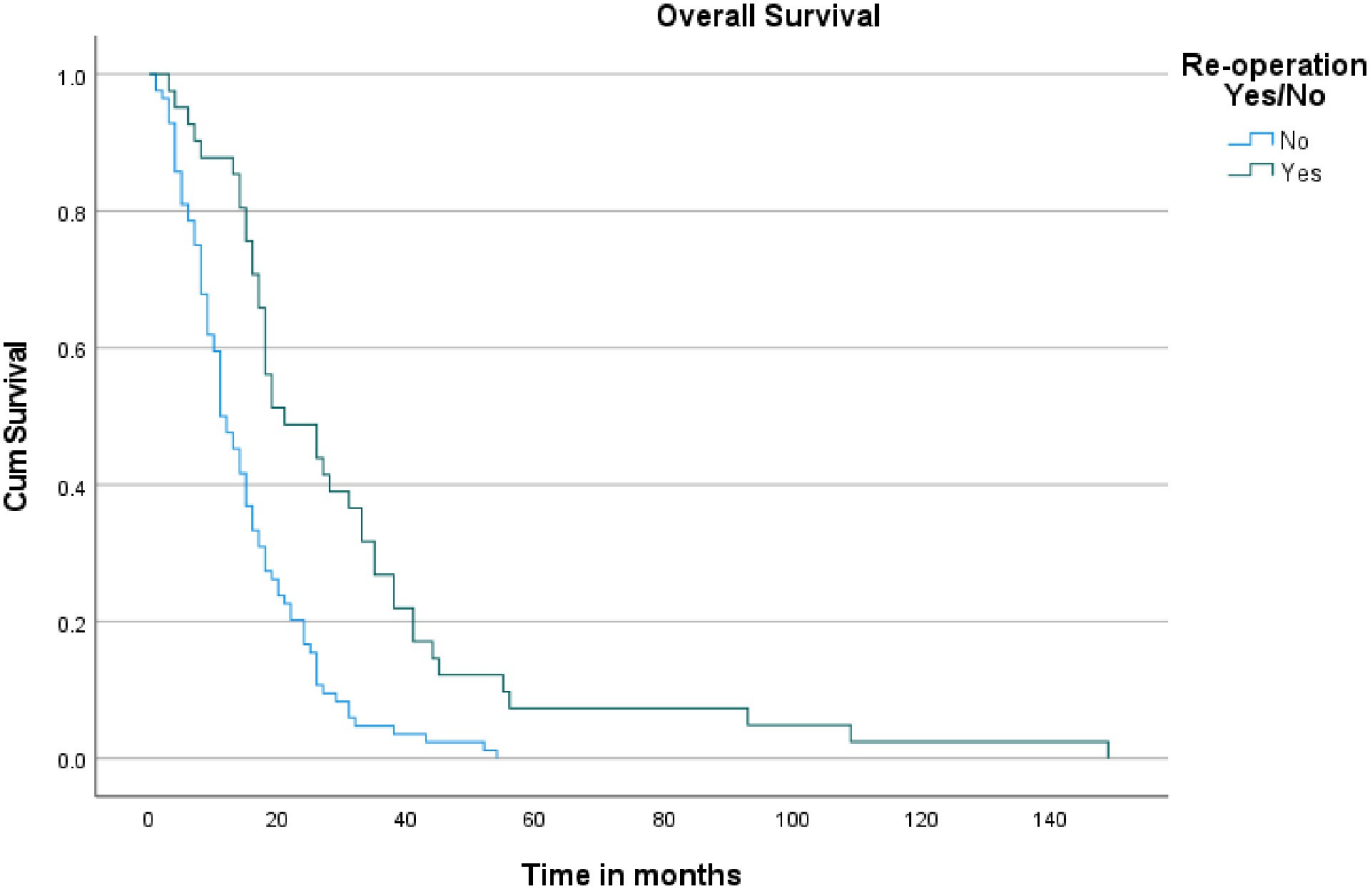
Kaplan-Meier curves for overall survival in patients undergoing initial resection vs. repeat resection.

Regarding PPS, the median survival time after progression was 5.9 months (95% CI: 2.145–9.589) for patients who underwent reoperation upon recurrence, compared to 5.1 months (95% CI: 3.425–6.775) for those who did not (log-rank p = 0.065) (Fig 2). As for OS, patients who underwent reoperation had a median survival of 21 months (95% CI: 10.963–31.037), which was significantly longer than the 11 months (95% CI: 8.605–13.395) observed for those who did not have a reoperation (log-rank p < 0.001) (Fig 3).

### Adjuvant treatment following recurrence

Following recurrence, 82 patients (80%) received adjuvant treatment, while 20 patients (20%) did not receive any further treatment. Among those who underwent adjuvant treatment, the modalities varied. Eighteen patients were treated with chemoradiotherapy (ChemoRT) combined with adjunct chemotherapy (Adj. CHT), and 17 patients received radiotherapy (RT) alone. Sixteen patients were administered temozolomide alone. Bevacizumab was used in 60 patients, and 17 patients received Lomustine. Other treatment options included Afinitor, Gefitinib, Irinotecan, Nivolumab, Etoposide, Panitumumab, and Regorafenib. The mean duration of adjuvant treatment after recurrence was 8 ±13 months.

### Complications following repeat resection

In the repeat resection group, the most common complications were pulmonary embolism and seizures, each occurring in 2 patients (Table 3). Other complications included weakness and cerebrospinal fluid (CSF) leak.

**Table 3:** Complications following repeat resection.

Complications following Repeat Resection	Number of patients
Pulmonary embolism	2
Seizures	2
Weakness	1
CSF leak	1

### Predictors of Overall Survival (OS)

The univariable Kaplan-Meier analysis identified several predictors of poor OS in patients with recurrent glioblastoma (Table 4). Gender did not significantly influence survival; however, the Charlson Comorbidity Index was a significant predictor of survival. Patients with an index of ≤3 had a median survival of 17 months (95% CI: 14.910–19.090) compared to 13 months (95% CI: 7.539–17.191) for those with an index >3, with a p-value of 0.038.

**Table 4:** Univariable Kaplan-Meier analysis of predictors for poor overall survival in patients with recurrent glioblastoma.

Variables	Median Survival in months (95% CI)	P-value
**Gender**		
Male	16 (13.235–18.765)	0.393
Female	15 (11.477–18.523)	
**Charlson Comorbidity Index**		
≤ 3	17 (14.910–19.090)	**0.038**
> 3	13 (7.539–17.191)	
**ASA Classification**		
< 3	16 (13.371–18.629)	**0.003**
≥ 3	11 (6.328–15.672)	
**Tumor Location (Lobes)**		
Temporal	14 (10.605–17.395)	0.314
Parietal	16 (7.684–24.316)	
Frontal	15 (11.136–18.864)	
Occipital	16 (.000–43.440)	
Other	12 (5.999–18.001)	
**Tumor Location (Side)**		
Right	15 (11.396–18.604)	**0.034**
Left	17 (14.306–19.694)	
Bilateral	4	
**Tumor Size**		
≤ 5 cm	15 (12.897–17.103)	0.661
< 5 cm	13 (8.357–17.643)	
**Tumor Focality**		
Unifocal	17 (15.111–18.889)	**<0.001**
Multifocal	6 (2.801–9.199)	
**Extent of Tumor Resection**		
GTR	18 (10.671–25.329)	**0.001**
Subtotal	11 (6.522–15.478)	
**IDH Mutational Status**		
Wildtype	14 (9.577–18.423)	0.433
Mutant	18 (13.596–22.404)	
**Ki67 Index**		
High	15 (12.685–17.315)	0.611
Low	15 (12.060–17.940)	
**Oncotherapy after the 1^st^ Surgery**		
Resection only	5 (3.868–6.132)	**0.021**
RT alone	14 (.000–28.761)	
Temodal or Avastin	4	
ChemoRT and Adj. CHT	16 (14.052–17.948)	
**Complications following 1^st^ Surgery**		
Yes	15 (6.235–23.765)	0.653
No	15 (12.992–17.008)	
**Location at Recurrence**		
Same	16 (13.620–18.380)	0.442
Different	9 (.000–19.802)	

ASA classification also had a significant impact on survival. Patients with an ASA classification of <3 had a median survival of 16 months (95% CI: 13.371–18.629), while those with an ASA classification of ≥3 had a median survival of 11 months (95% CI: 6.328–15.672), with a p-value of 0.003. Tumor location within the brain lobes resulted in comparable survival outcomes: patients with temporal lobe tumors, parietal lobe tumors, frontal lobe tumors, occipital lobe tumors, and those with tumors in other locations had a median survival of 14 months (95% CI: 10.605–17.395), 16 months (95% CI: 7.684–24.316), (95% CI: 11.136–18.864), 16 months (95% CI: 0.000–43.440), and 12 months (95% CI: 5.999–18.001), respectively (p = 0.314).

Side-specific tumor location demonstrated that patients with right-sided tumors had a median survival of 15 months (95% CI: 11.396–18.604), compared to 17 months (95% CI: 14.306–19.694) for those with left-sided tumors and 4 months for those with bilateral tumors, with a significant p-value of 0.034. Tumor focality was a strong predictor of survival, as patients with unifocal tumors had a median survival of 17 months (95% CI: 15.111–18.889), while those with multifocal tumors had a median survival of only 6 months (95% CI: 2.801–9.199), with a highly significant p-value of <0.001.

The extent of tumor resection following the first surgery significantly influenced survival, with an initial gross total resection (GTR) resulting in a median survival of 18 months (95% CI: 10.671–25.329) compared to 11 months (95% CI: 6.522–15.478) for subtotal resection (p = 0.001). IDH mutational status, as well as Ki-67 index levels, did not influence survival. Oncotherapy after the first surgery was a significant factor affecting survival. Patients undergoing resection only had a median survival of 5 months (95% CI: 3.868–6.132), while those receiving radiotherapy alone had a median survival of 14 months (95% CI: 0.000–28.761). Conversely, those treated with chemoradiotherapy, and adjuvant chemotherapy (STUPP protocol) had a median survival of 16 months (95% CI: 14.052–17.948), with a significant p-value of 0.021.

Post-surgery complications did not significantly affect survival. Both groups, those with and without complications, had a median survival of 15 months (95% CI: 6.235–23.765 for those with complications and 12.992–17.008 for those without) (p = 0.653). There was no difference in survival based on the location of recurrence (p = 0.442).

In the multivariable Cox regression analysis, several factors were evaluated for their impact on overall survival in patients with recurrent glioblastoma (Table 5). Tumor focality emerged as a significant predictor, with multifocal tumors associated with poorer survival compared to unifocal tumors (HR: 0.507, 95% CI: 0.274–0.935, p = 0.030). Oncotherapy after the first surgery also demonstrated a strong impact on survival. Patients who underwent radiotherapy alone had significantly worse outcomes (HR: 7.032, 95% CI: 2.378–20.798, p<0.001) compared to those who received the STUPP protocol. The extent of tumor resection showed a trend towards improved survival with gross total resection (GTR), though this was not statistically significant (HR: 0.663, 95% CI: 0.430–1.022, p = 0.063). Other variables, such as the Charlson Comorbidity Index and ASA classification, did not show significant associations with survival after adjusting for confounding factors.

**Table 5:** Predictors of poor overall survival in patients with recurrent glioblastoma based on multivariable Cox regression analysis (N = 125 patients).

Variable	HR	95% confidence interval	P Value
**Charlson Comorbidity Index**	0.808	(0.526–1.241)	0.330
**ASA Classification**	0.630	(0.377–1.054)	0.252
**Tumor Location (Side)**			
Right	Ref		0.085
Left	0.404	(0.084–1.946)	0.259
Bilateral	0.281	(0.062–1.283))	0.101
**Tumor Focality**	0.507	(0.274–0.935)	**0.030**
**Oncotherapy after the 1^st^ Surgery**			
Resection only	Ref		**0.003**
RT alone	7.032	(2.378–20.798)	**<0.001**
Temodal or Avastin	1.804	(0.744–4.370)	0.909
ChemoRT and Adj. CHT	6.171	(0.695–54.778)	0.999
**Extent of Tumor Resection**	0.663	(0.430–1.022)	0.063

## Discussion

Based on the literature presented in Table 6, we presented a detailed comparison of outcomes between patients with recurrent GBM who underwent reoperation and those who did not. Reoperation for recurrent GBM is generally associated with a better PFS compared to no reoperation. For instance, studies such as those by Quick et al. and Rusthoven et al. reported PFS rates of 13.0 and 21.8 months after reoperation, respectively. This is notably longer than the PFS rates observed in non-reoperation cohorts, which generally ranged from 5.3 to 9.0 months. In studies such as that by McNamara et al., PFS after reoperation was reported at 7.1 months, while the non-reoperation PFS rate was significantly shorter at 9.9 months. These findings suggest that reoperation can effectively delay disease progression in recurrent GBM patients, potentially due to the removal of tumor bulk and reduced tumor burden, which may help prolong the time before disease progression. However, the variability in PFS results among studies indicates that the benefit of reoperation can be influenced by factors such as the extent of resection, the patient’s overall health, and the timing of the surgery. For example, studies such as those by Park et al. and Chen et al. reported varying PFS outcomes depending on the reoperation approach and patient subgroup characteristics.

**Table 6:** A literature review of reoperation on recurrent glioblastoma.

*Author*	GBM patients who underwent initial surgery	Reoperation on recurrent GBM	Median time between first and second surgery (months)	PFS after repeat surgery (months)	OS with redo- surgery (months)	OS without redo- surgery (months)
*Franceschi et al.* [17]	232	102 (44%)	13.1	9.6	25.8	18.6
*Quick et al.* [18]	40	40 (100%)	10.2 (early if < 10 and late if > 10)	13.0	26.2	16.2
*McNamara et al.* [19]	584	107 (18.3%)	11.5	7.1	20.9	9.9
*Park et al.* [20]	55	55 (100%)	10.0	13.0	GP: 20.0; IP: 18.0; PP: 4.0	GP: 14.0; IP: 8.0; PP: 3.0
*Terasaki et al.* [21]	35	7 (20%)	6.9	9.0	15.1	-
*Voisin et al.* [22]	174	87 (50%)	-	10.5	30.6	18.5
*Rusthoven et al.* [23]	34	34 (100%)	6.7	21.8	30.2	-
*Harsh et al.* [24]	39	39 (100%)	11.2	8.4	19.1	-
*De Bonis et al.* [25]	36	17 (47.2%)	-	6.0 vs 5.0	-	-
*Ma et al.* [26]	205	52 (25.3%)	-	-	16.0	10.7
*Azoulay et al.* [27]	183	69 (38%)	7.43	9.6 vs 5.3	-	-
*Chen et al.* [28]	65	20 (30.7%)	6.3	13.5 vs 5.8	25.4	11.6
*Woernle et al.* [29]	98	40 (40.8%)	-	GP: 13.93; IP: 8.33	18.9	14.81
*Tully et al.* [14]	204	40 (24%)	-	8.3 vs 6.7	20.1	9.0
*Helseth et al.* [30]	516	65 (12.6%)	7.0	-	18.4	8.6

PFS = Progression-free survival

OS = Overall survival

GP, IP, PP, = Good prognosis, intermediate prognosis, poor prognosis

Moreover, reoperation generally has a positive impact on OS, but the extent of this benefit can vary. For example, Voisin et al. and Quick et al. reported OS with reoperation ranging from 18.4 to 30.6 months, which is substantially longer than OS without reoperation, which ranges from 8.6 to 18.6 months. This extended survival time observed in patients undergoing reoperation suggests that surgical intervention can offer a significant survival advantage by providing symptomatic relief, reducing tumor burden, and potentially improving the efficacy of subsequent treatments. Conversely, in studies such as those by Ma et al. and Azoulay et al., where direct comparisons of OAS with and without reoperation are less clear, the available data suggest that while reoperation does contribute to improved survival, the benefit might be less pronounced or variable depending on individual patient factors and the specific treatment protocols used.

Overall, reoperation for recurrent GBM appears to offer substantial benefits in terms of both PFS and OS compared to non-reoperation, with PFS improvements ranging from a few months to over a year and OS improvements similarly varying. The variability in outcomes highlights the importance of personalized treatment planning. Factors such as tumor location, previous treatment history, patient health, and the specifics of the surgical approach play crucial roles in determining the effectiveness of reoperation.

Given these findings, it is essential for clinicians to carefully consider individual patient circumstances when deciding on the management of recurrent GBM. While reoperation can provide significant benefits, especially in extending PFS and OS, the decision should be guided by a comprehensive evaluation of potential risks, benefits, and patient-specific factors to optimize overall treatment outcomes.

Our institution’s data on reoperation for recurrent GBM reveals significant findings regarding survival and outcomes. Among the 125 patients analyzed, 41 underwent repeat resection, while 84 had only a single resection. The median PFS for patients who underwent repeat resection was 15.9 months, which was significantly longer than the 6.7 months observed in those who did not undergo reoperation, with a p-value of <0.001 indicating a highly significant difference. The 95% confidence interval for the PFS in the repeat resection group was not provided, but the significant p-value underscores a robust extension in time before disease progression.

Regarding OS, patients who underwent repeat resection had a median survival of 21 months, compared to 11 months for those who did not, with a p-value of <0.001, reflecting a substantial improvement in survival. Although the 95% confidence interval for the OS in the repeat resection group was not specified, the statistically significant p-value highlights the clear survival advantage associated with reoperation. Regarding PPS, the median survival after progression was 5.9 months for patients who had repeat resection, versus 5.1 months for those who did not, with a p-value of 0.065. This p-value suggests a trend toward improved PPS with reoperation. However, the improvement was not statistically significant.

The extent of complications following initial surgery was notably lower in the repeat resection group, with a complication rate of 4.9% compared to 21.4% in the single resection group, yielding a p-value of 0.018, which indicates a significant reduction in complications. Common complications in the repeat resection group included pulmonary embolism and seizures, but these were relatively infrequent. The most frequent post-surgical complications in the single resection cohort included deep vein thrombosis, hemiparesis/weakness, and seizures. Survival outcomes further revealed that patients with unifocal tumors had a median survival of 17 months (95% CI: 15.111–18.889), whereas those with multifocal tumors had a significantly shorter median survival of 6 months (95% CI: 2.801–9.199), with a highly significant p-value of <0.001.

The extent of initial tumor resection also impacted survival, with gross total resection (GTR) yielding a median survival of 18 months (95% CI: 10.671–25.329) compared to 11 months (95% CI: 6.522–15.478) for subtotal resection, with a p-value of 0.001. Additionally, patients who received the STUPP protocol (chemoradiotherapy and adjuvant chemotherapy) had a median survival of 16 months (95% CI: 14.052–17.948), compared to 5 months (95% CI: 3.868–6.132) for those who underwent resection only, indicating a significant improvement in survival associated with comprehensive adjuvant treatment, with a p-value of 0.021. Complications did not significantly affect OS, with both groups having a median survival of 15 months (95% CI: 6.235–23.765 for those with complications and 12.992–17.008 for those without) and a p-value of 0.653. Our data clearly indicate that reoperation for recurrent glioblastoma offers significant benefits in extending PFS and OS while demonstrating variability in outcomes based on factors such as tumor focality, the extent of resection, and adjuvant treatments.

The findings from our institution align closely with findings from the broader literature, underscoring the significant benefits of repeat surgical intervention. The median PFS for patients who underwent repeat resection was 15.9 months, which is consistent with the range of PFS improvements reported in previous literature regarding reoperation. For example, Quick et al. and Rusthoven et al. reported PFS rates of 13.0 months and 21.8 months, respectively. The statistically significant p-value of <0.001 in our data further supports the assertion that repeat resection can effectively extend disease control before progression. Similarly, our median OS of 21 months for patients who underwent repeat resection reflects the substantial survival benefit noted in studies such as those by Quick et al., who observed survival ranges up to 30.6 months. The p-value of <0.001 in our study highlights the clear advantage of reoperation in prolonging OS. While our median PPS of 5.9 months showed a trend towards improvement over the 5.1 months observed in non-reoperation patients, the lack of statistical significance (p = 0.065) is in line with literature findings, which often report variable impacts of reoperation on PPS.

The significant reduction in complications following initial surgery in our repeat resection cohort (4.9% vs. 21.4%, p = 0.018) supports literature observations that reoperation can be associated with fewer complications compared to the initial surgery. Furthermore, our data on tumor focality, with unifocal tumors having a median survival of 17 months and multifocal tumors having a median survival of only 6 months, aligns with literature findings indicating that tumor characteristics significantly influence survival outcomes. The impact of the extent of initial tumor resection on survival, with gross total resection correlating with longer survival (18 months vs. 11 months for subtotal resection), is consistent with literature reports emphasizing the importance of complete surgical resection. Additionally, the survival advantage associated with the STUPP protocol in our study, showing a median survival of 16 months for those receiving comprehensive adjuvant treatment, is consistent with the consensus found in previous literature reports on the efficacy of combined chemoradiotherapy and adjuvant chemotherapy.

The decision to reoperate is complex and must also consider factors like comorbidities and duration of initial PFS. Patients with significant comorbidities or poor functional status may not tolerate surgery well, making it less feasible. Additionally, a longer initial PFS might indicate a more indolent tumor biology, making reoperation more likely to provide further benefit. Overall, our findings corroborate existing literature on the benefits of reoperation for recurrent GBM, emphasizing the importance of surgical intervention in extending disease control and OS, while also highlighting the consistency in observed trends and outcomes across different studies.

### Limitations

While our findings provide valuable insights into the outcomes of reoperation for recurrent GBM, several limitations must be considered. First, the complexity of patient selection for reoperation introduces potential selection bias. Patients selected for repeat resection are often younger, in better overall health, and have less extensive disease, factors that may independently contribute to improved outcomes irrespective of the surgical intervention.

Second, the retrospective nature of our data collection and analysis introduces inherent biases and constraints, including variability in surgical techniques and adjuvant treatment protocols over time. Furthermore, while our institutional data align with broader trends reported in the literature, the single-center design limits the generalizability of our findings to other populations and healthcare settings. Finally, future studies should investigate the influence of molecular and genetic tumor profiles on the outcomes of reoperation, as these factors are increasingly important in tailoring individualized treatment strategies.

## Conclusion

Based on the data from our patient cohort, we consider re-do surgery for patients with recurrent glioblastoma (GBM) in select cases. Specifically, reoperation should be prioritized in situations where the recurrent lesion is located in a non-eloquent region of the brain, as the risk of significant neurological deficits is minimized. Additionally, reoperation is advised for lesions that are easily accessible surgically, allowing for safer and more effective resections with reduced operative risk. Another key consideration is the presence of mass effect or associated edema, as surgical resection in these cases can significantly reduce intracranial pressure, alleviating symptoms such as headaches, neurological deficits, and seizures. By decreasing mass effect and edema, reoperation has the potential to enhance patients’ quality of life and provide symptomatic relief, which is a critical factor in managing recurrent GBM.

## References

[1] Louis DN, Perry A, Wesseling P, et al. The 2021 WHO Classification of Tumors of the Central Nervous System: a summary. *Neuro Oncol*. 2021;23(8):1231–1251. doi: 10.1093/neuonc/noab106 34185076 PMC8328013

[2] Deorah S, Lynch CF, Sibenaller ZA, Ryken TC. Trends in brain cancer incidence and survival in the United States: Surveillance, Epidemiology, and End Results Program, 1973 to 2001. Neurosurgical focus 2006;20(4):E1. doi: 10.3171/foc.2006.20.4.E1 16709014

[3] Stupp R, Mason WP, Van Den Bent MJ, Weller M, Fisher B, Taphoorn MJ, et al. Radiotherapy plus concomitant and adjuvant temozolomide for glioblastoma. N Engl J Med 2005;352(10):987–996. doi: 10.1056/NEJMoa043330 15758009

[4] Boiardi A, Silvani A, Eoli M, Lamperti E, Salmaggi A, Gaviani P, et al. Treatment of recurrent glioblastoma: can local delivery of mitoxantrone improve survival? J Neurooncol 2008;88:105–113. doi: 10.1007/s11060-008-9540-6 18283418

[5] Sampson JH. Alternating electric fields for the treatment of glioblastoma. JAMA 2015;314(23):2511–2513. doi: 10.1001/jama.2015.16701 26670969

[6] Macdonald DR, Cascino TL, Schold SC Jr, Cairncross JG. Response criteria for phase II studies of supratentorial malignant glioma. Journal of clinical oncology 1990;8(7):1277–1280. doi: 10.1200/JCO.1990.8.7.1277 2358840

[7] Wen PY, Macdonald DR, Reardon DA, Cloughesy TF, Sorensen AG, Galanis E, et al. Updated response assessment criteria for high-grade gliomas: response assessment in neuro-oncology working group. Journal of clinical oncology 2010;28(11):1963–1972. doi: 10.1200/JCO.2009.26.3541 20231676

[8] Hervey-Jumper SL, Berger MS. Reoperation for recurrent high-grade glioma: a current perspective of the literature. Neurosurgery 2014;75(5):491–499. doi: 10.1227/NEU.0000000000000486 24991712

[9] Niranjan A, Kano H, Iyer A, Kondziolka D, Flickinger JC, Lunsford LD. Role of adjuvant or salvage radiosurgery in the management of unresected residual or progressive glioblastoma multiforme in the pre–bevacizumab era. J Neurosurg 2015;122(4):757–765. doi: 10.3171/2014.11.JNS13295 25594327

[10] Bianconi A, Koumantakis E, Gatto A, et al. Effects of Levetiracetam and Lacosamide on survival and seizure control in IDH-wild type glioblastoma during temozolomide plus radiation adjuvant therapy. *Brain Spine*. 2023;4:102732. Published 2023 Dec 14. doi: 10.1016/j.bas.2023.102732 38510602 PMC10951696

[11] Stark AM, Hedderich J, Held-Feindt J, Mehdorn HM. Glioblastoma—the consequences of advanced patient age on treatment and survival. Neurosurg Rev 2007;30:56–62. doi: 10.1007/s10143-006-0051-7 17119901

[12] Pool LJ. The Management of Recurrent Gliomas: Chapter XI. Neurosurgery 1968;15:265–287. 10.1093/neurosurgery/15.cn_suppl_1.265 4331143

[13] Chaichana KL, Zadnik P, Weingart JD, Olivi A, Gallia GL, Blakeley J, et al. Multiple resections for patients with glioblastoma: prolonging survival. J Neurosurg 2013;118(4):812–820. doi: 10.3171/2012.9.JNS1277 23082884 PMC3700339

[14] Tully PA, Gogos AJ, Love C, Liew D, Drummond KJ, Morokoff AP. Reoperation for recurrent glioblastoma and its association with survival benefit. Neurosurgery 2016;79(5):678–689. doi: 10.1227/NEU.0000000000001338 27409404

[15] Park JK, Hodges T, Arko L, Shen M, Iacono DD, McNabb A, et al. Scale to predict survival after surgery for recurrent glioblastoma multiforme. Journal of clinical oncology 2010;28(24):3838. doi: 10.1200/JCO.2010.30.0582 20644085 PMC2940401

[16] Cofano F, Bianconi A, De Marco R, et al. The Impact of Lateral Ventricular Opening in the Resection of Newly Diagnosed High-Grade Gliomas: A Single Center Experience. *Cancers (Basel)*. 2024;16(8):1574. Published 2024 Apr 19. doi: 10.3390/cancers16081574 38672655 PMC11049264

[17] Franceschi E, Bartolotti M, Tosoni A, Bartolini S, Sturiale C, Fioravanti A, et al. The effect of re-operation on survival in patients with recurrent glioblastoma. Anticancer Res 2015;35(3):1743–1748. 25750337

[18] Quick J, Gessler F, Dützmann S, Hattingen E, Harter PN, Weise LM, et al. Benefit of tumor resection for recurrent glioblastoma. J Neurooncol 2014;117:365–372. doi: 10.1007/s11060-014-1397-2 24535317

[19] McNamara MG, Lwin Z, Jiang H, Templeton AJ, Zadeh G, Bernstein M, et al. Factors impacting survival following second surgery in patients with glioblastoma in the temozolomide treatment era, incorporating neutrophil/lymphocyte ratio and time to first progression. J Neurooncol 2014;117:147–152. doi: 10.1007/s11060-014-1366-9 24469854

[20] Park C, Kim JH, Nam D, Kim C, Chung S, Kim Y, et al. A practical scoring system to determine whether to proceed with surgical resection in recurrent glioblastoma. Neuro-oncology 2013;15(8):1096–1101. doi: 10.1093/neuonc/not069 23800677 PMC3714158

[21] Terasaki M, Ogo E, Fukushima S, Sakata K, Miyagi N, Abe T, et al. Impact of combination therapy with repeat surgery and temozolomide for recurrent or progressive glioblastoma multiforme: a prospective trial. Surg Neurol 2007;68(3):250–254. doi: 10.1016/j.surneu.2006.11.042 17719957

[22] Voisin MR, Zuccato JA, Wang JZ, Zadeh G. Surgery for Recurrent Glioblastoma Multiforme: A Retrospective Case Control Study. World Neurosurgery 2022;166:e624–e631. doi: 10.1016/j.wneu.2022.07.070 35870781

[23] Rusthoven KE, Olsen C, Franklin W, Kleinschmidt-DeMasters BK, Kavanagh BD, Gaspar LE, et al. Favorable prognosis in patients with high-grade glioma with radiation necrosis: the University of Colorado reoperation series. International Journal of Radiation Oncology* Biology* Physics 2011;81(1):211–217. doi: 10.1016/j.ijrobp.2010.04.069 20732762

[24] Harsh IV GR, Levin VA, Gutin PH, Seager M, Silver P, Wilson CB. Reoperation for recurrent glioblastoma and anaplastic astrocytoma. Neurosurgery 1987;21(5):615–621. doi: 10.1227/00006123-198711000-00002 2827052

[25] De Bonis P, Fiorentino A, Anile C, Balducci M, Pompucci A, Chiesa S, et al. The impact of repeated surgery and adjuvant therapy on survival for patients with recurrent glioblastoma. Clin Neurol Neurosurg 2013;115(7):883–886. doi: 10.1016/j.clineuro.2012.08.030 22959214

[26] Ma X, Lv Y, Liu J, Wang D, Huang Q, Wang X, et al. Survival analysis of 205 patients with glioblastoma multiforme: clinical characteristics, treatment and prognosis in China. Journal of Clinical Neuroscience 2009;16(12):1595–1598. doi: 10.1016/j.jocn.2009.02.036 19793663

[27] Azoulay M, Santos F, Shenouda G, Petrecca K, Oweida A, Guiot MC, et al. Benefit of re-operation and salvage therapies for recurrent glioblastoma multiforme: results from a single institution. J Neurooncol 2017;132:419–426. doi: 10.1007/s11060-017-2383-2 28374095

[28] Chen MW, Morsy AA, Liang S, Ng WH. Re-do craniotomy for recurrent grade IV glioblastomas: impact and outcomes from the National Neuroscience Institute Singapore. World neurosurgery 2016;87:439–445. doi: 10.1016/j.wneu.2015.10.051 26585720

[29] Woernle CM, Péus D, Hofer S, Rushing EJ, Held U, Bozinov O, et al. Efficacy of surgery and further treatment of progressive glioblastoma. World neurosurgery 2015;84(2):301–307. doi: 10.1016/j.wneu.2015.03.018 25797075

[30] Helseth R, Helseth E, Johannesen TB, Langberg CW, Lote K, Rønning P, et al. Overall survival, prognostic factors, and repeated surgery in a consecutive series of 516 patients with glioblastoma multiforme. Acta Neurol Scand 2010;122(3):159–167. doi: 10.1111/j.1600-0404.2010.01350.x 20298491

